# Microbiota‐Microglia Interactions Modulate Beta‐Amyloid Levels

**DOI:** 10.1002/alz.087775

**Published:** 2025-01-03

**Authors:** Laura M Cox

**Affiliations:** ^1^ Harvard Medical School, Brigham and Women’s Hospital, Boston, MA USA

## Abstract

**Background:**

The gut microbiota and microglia play critical roles in Alzheimer’s disease (AD), and more information is needed on how specific bacteria affect brain immunity in AD. We found that *Bacteroides* increased in aging Tg2576 mice and correlated with amyloid‐beta (Aβ) plaques and hypothesized that *Bacteroides* worsens AD pathology by modulating microglia. *Bacteroides* is also elevated in AD patients and correlates with cerebrospinal fluid levels of Aβ and tau.

**Method:**

To test the effect of *B. fragilis* on Aβ plaques and microglia, we administered *Bacteroides fragilis* to APP/PS1‐21 mice, assessed plaques by immunohistochemistry, and investigated microglial transcriptional responses on FAC sorted microglia by RNAseq. To determine whether B. fragilis suppressed microglial uptake, we also administered B. fragilis to aged WT mice for two months, then injected fluorescently labeled human Aβ1‐42 peptides to the brain and measured FITC‐Aβ by flow cytometry.

**Result:**

We found that *B. fragilis* increased Aβ plaques in female mice, modulated cortical gene expression involved in amyloid processing, and down regulated expression of microglial genes involved in phagocytosis and protein degradation. To investigate microglial phagocytic function, we administered *B. fragilis* to aged WT female and male mice for 2 months, injected fluorescently labeled human Aβ1‐42 peptides to the brain, and found that *B. fragilis* suppressed uptake of Aβ in both male and female mice. Depleting murine *Bacteroidota* with metronidazole decreased amyloid load in aged 5xFAD mice, and activated microglial pathways related to phagocytosis, cytokine signaling, and lysosomal degradation.

**Conclusion:**

Taken together, these data suggest that bacteria in the *Bacteroidota* phylum contribute to AD pathogenesis by suppressing microglia phagocytic function, leading to impaired Aβ clearance and accumulation of plaques.

